# Comparison of Surti goat milk with cow and buffalo milk for physicochemical characteristics, selected processing-related parameters and activity of selected enzymes

**DOI:** 10.14202/vetworld.2017.477-484

**Published:** 2017-05-04

**Authors:** Darshna B. Prajapati, Dharti B. Kapadiya, Amit Kumar Jain, Bhavbhuti M. Mehta, Vijaykumar B. Darji, Kishorkumar D. Aparnathi

**Affiliations:** 1 Department of Dairy Chemistry, SMC College of Dairy Science, Anand Agricultural University, Anand, Gujarat, India; 2 Department of Agricultural Statistics, BA College of Agriculture, Anand Agricultural University, Anand, Gujarat, India

**Keywords:** heat coagulation time, lipase activity, physicochemical characteristics, proteolytic activity, rennet coagulation time

## Abstract

**Aim:**

The study was undertaken to find out the physicochemical characteristics, selected processing-related parameters and activity of selected enzymes in Surti goat milk.

**Materials and Methods:**

Milk samples from Surti goats and buffalo milk samples were collected during the period from July 2013 to January 2014 at Reproductive Biology Research Unit, Anand Agricultural University (AAU), Anand. Milk samples from Kankrej cows were collected from Livestock Research Station, AAU, Anand. Samples were analyzed for physicochemical characteristics such as acidity, viscosity, surface tension, specific gravity, refractive index, freezing point, and electrical conductivity. Samples were also analyzed for selected processing-related parameters such as heat coagulation time (HCT), rennet coagulation time (RCT), rate of acid production by starter culture, alcohol stability, and activity of selected enzymes such as alkaline phosphatase activity, catalase activity, proteolytic activity, and lipase activity.

**Results:**

Goat milk had the highest acidity, viscosity and surface tension, followed by cow milk and buffalo milk. However, the differences in acidity, specific gravity, surface tension, refractive index, electrical conductivity, HCT and lipase activity of three types of milk studied, *viz*., goat, cow, and buffalo milk were found statistically non-significant (p<0.05). The buffalo milk had the highest specific gravity, followed by those found in cow and goat milk. The viscosity, freezing point and RCT of goat milk was significantly lower (p>0.05) than that of the buffalo milk. However, the difference in viscosity, freezing point and RCT of goat milk and that of the cow milk was statistically non-significant. The cow milk had the highest refractive index, followed by goat and buffalo milk. The cow milk had the highest proteolytic activity and heat coagulation time (HCT), followed by those found in buffalo and goat milk. The goat milk had the lowest freezing point, lipase activity, and RCT, followed by those found in cow and buffalo milk. The goat milk had the highest electrical conductivity, followed by those found in buffalo and cow milk. The collected goat, cow and buffalo milk samples showed negative stability at 68% (v/v) alcohol concentration. Goat milk showed positive alcohol test at 75% (v/v) alcohol concentration. Acidity was found to increase proportionally with time. After 14 h, it was found that goat milk became thicker, but the curd had a very low consistency. Cow milk had the highest alkaline phosphatase activity and catalase activity followed by those found in goat milk and lowest alkaline phosphatase activity and catalase activity was found in buffalo milk. The alkaline phosphatase activity and proteolytic activity of goat milk was significantly lower (p>0.05) than that of the cow milk. However, the difference in alkaline phosphatase activity and proteolytic activity of goat milk and that of the buffalo milk was statistically non-significant. Alkaline phosphatase activity of buffalo milk was significantly lower (p>0.05) than that of the alkaline phosphatase activity in cow milk.

**Conclusion:**

It can be concluded from the study that the goat milk has highest acidity, viscosity, electrical conductivity, and surface tension compared to that of cow and buffalo milk. The goat milk has lowest specific gravity, freezing point, proteolytic activity, lipase activity, RCT and HCT compared to cow and buffalo milk. Goat milk had highest refractive index compared to buffalo milk, whereas lowest refractive index compared to cow milk. Goat milk showed positive alcohol test at 75% (v/v) alcohol concentration. The curd formed from goat milk after 14 h was having very weak consistency. The goat milk has higher alkaline phosphatase activity, catalase activity compared to buffalo milk while it has lower alkaline phosphatase activity, catalase activity compared to cow milk.

## Introduction

The milk composition of dairy animals has been widely studied worldwide and thousands of references are available especially with regard to milk consumed by humans. The literature is more related to cow and buffalo milk than goat and sheep milk. Goat’s milk offers a wide variety of health benefits due to its low fat, low lactose, calcium richness, antioxidant and antimicrobial properties [1-3]. Goat milk also has natural anti-inflammatory, bioavailability, antimucosal and ultra-nourishing properties. Goat milk provides a healthy and a balanced diet for those who are allergic to cow milk [4]. Information on composition and physicochemical characteristics of goat milk is essential for successful development of dairy goat farming as well as for marketing the goat milk products [5]. Goat milk has a stronger flavor due to the liberation of short-chain fatty acids, which gives a goatee smell [6,7].

Goat milk is more digestible because of its small-sized fat globules and uniform protein and fat distribution. Modified goat milk can also be used in baby feeding [8]. The nutritional value of milk is closely related to its composition, which is highly affected by factors such as breed, feed, stage of lactation, and season [9-11]. Goat milk is a good source of calcium, phosphorus, potassium, magnesium, sodium, and iron [1,3]. The various processing-related parameters such as alcohol test (indicator of milk freshness and propensity of milk for heat coagulation), heat stability (time required to coagulate upon heating), and rennet coagulation time (RCT) (time required to coagulate the milk by enzyme) are very useful during processing of milk and preparation of milk products, thereby setting up the standard procedures for the preparation of products from goat milk. Goat milk has shorter RCT, less resistance to heat treatment, weaker curd and low cheese yields which explain the significant differences from cow and other milk in digestion by infants and patients which traditionally have been explained by the “homogenized” nature of goat milk fat [12,13]. In spite of these important parameters, no work so far has been carried out in Gujarat.

The world’s goat population was around 867 million in 2009, with over 60% of that found in Asia and more than 95% in developing countries of the world. China has the largest goat herd with 195.6 million, followed by India with 120.0 million, and Pakistan 56.7 million. Gujarat goat milk production (1000 tons) during the year 2007-2008, 2009-2010, and 2010-2011 was 248, 231, and 236, respectively [14]. The value of goat milk in human nutrition has so far received very little factual and academic attention despite its medical need for some people especially infants afflicted with various ailments, including cow milk protein sensitivities [15,16].

The Food Safety and Standards Authority of India, Ministry of Health and Family Welfare, and Government of India were interested to develop new standards of milk and milk products. The data on physicochemical characteristics, selected processing-related parameters, and activity of selected enzymes and related information of milk of various species and breed of animals were lacking. Moreover, there were no reported values on these aspects of goat milk in Anand, Gujarat. Therefore, there was a need to undertake systematic study to generate data. This work provides basic database for physicochemical characteristics, selected processing-related parameters, and activity of selected enzymes of Surti goat milk. Moreover, the selected characteristics such as acidity, viscosity, electrical conductivity, surface tension, specific gravity, and freezing point as well as enzymatic activities (e.g., proteolytic and lipase activity), HCT and RCT of Surti goat were compared with cow and buffalo milk. This information will be beneficial to goat keepers, industrial personnel, and various government agencies as well as to society.

## Materials and Methods

### Ethical approval

Ethical approval was not applicable for this study. However, milk samples were collected as per standard milking techniques.

### Milk samples

Goat milk samples of Surti breed and buffalo milk samples were collected during the period between July 2013 and January 2014 at Reproductive Biology Research Unit, Anand Agricultural University (AAU), Anand, Gujarat, India. Cow milk samples of Kankrej breed were collected from Livestock Research Station, AAU, Anand, Gujarat, India, and used for investigation. Raw milk samples were collected at milking time in a clean and dry container. The samples were transported to the laboratory. Five replications of each goat, buffalo, and cow milk were carried out in total.

### Chemicals

During the entire study, chemicals used for chemical analysis for various properties were of analytical grade.

### Physicochemical characteristics

The acidity, specific gravity, viscosity (by Oswald viscometer), surface tension (by stalagmometer) of all milk samples were determined by method as described in BIS Handbook [17]. The refractive index, freezing point and electrical conductivity of goat, cow and buffalo milk were determined by Ultrasonic Milk Analyzer.

### Selected processing-related parameters

Alcohol test was determined as per the procedure described by BIS Handbook [17]. HCT was determined in a thermostatically controlled oil bath at 140°C according to the method of Muir and Sweetsur [18]. For the determination of RCT, a macro film technique was used (developed by Gallager and Mulvihill [19]). Rate of acid production was done by the method of Ahmad *et al*. [20]. The rate of acid production in milk was measured by adding starter culture of *Streptococcus thermophilus* in milk at the rate of 2% of milk and incubated at 37°C. The development of acidity was measured at every 2 h intervals.

### Activity of selected enzymes

Alkaline phosphatase activity was determined by Sharma [21]. Catalase activity was determined by the method of Karmakar *et al*. [22]. One catalase unit is expressed as ml of 0.1 N sodium thiosulfate required for control determination less the titration value for the experimental solution per 5 ml of the enzyme solution. Lipase activity was determined by the method of Kumar *et al*. [23]. One lipase unit is equivalent to a preparation which could liberate fatty acids requiring 1 ml of 0.01 N alcoholic KOH with 1 h incubation period at 37°C. Proteolytic activity was measured by the method of Gamble *et al*. [24].

### Statistical analysis

The collected data were subjected to statistical analysis. Data were analyzed by completely randomized design and critical difference test at 5% level of significance (p<0.05) as per the procedure mentioned by Rudolf *et al*. [25].

## Results

The physicochemical characteristics of goat, cow and buffalo milk are mentioned in Table-1. The acidity range in goat milk was between 0.132% and 0.196% lactic acid (LA), and the mean value was 0.164% LA. The acidity in cow milk ranged between 0.115% and 0.159% LA and the mean value was 0.137% LA. On the other hand, the acidity range in buffalo milk was between 0.103% and 0.157% LA and the mean value was 0.130% LA. Thus, goat milk had the highest acidity, followed by those found in cow milk and lowest acidity observed in buffalo milk. However, the differences in acidity of three types of milk studied, *viz*., goat, cow and buffalo milk were found statistically non-significant (p<0.05). The range of specific gravity of goat milk determined in five replications was 1.028-1.032 and the mean value was 1.030. Similarly, the specific gravity range in cow milk was between 1.030 and 1.032 and the mean value was 1.031. On the other hand, specific gravity range in buffalo milk was between 1.030 and 1.034 and the mean value was 1.032. The buffalo milk had the highest specific gravity, followed by those found in cow and goat milk. However, the differences in specific gravity of three types of milk studied, *viz*., goat, cow and buffalo milk were found statistically non-significant (p<0.05). The range of viscosity in goat milk was between 1.44 and 1.62 cp and the mean value was 1.53 cp. The viscosity range in cow milk was between 1.53 and 1.65 cp and the mean value was 1.59 cp. Buffalo milk had ­viscosity between 1.68 and 1.90 cp and the mean value was 1.79 cp. Thus, goat milk had the lowest viscosity, which was followed by cow milk and highest viscosity observed in buffalo milk. The viscosity of goat milk was significantly lower than that of the buffalo milk. However, the difference in viscosity of goat milk and that of the cow milk was statistically non-significant. The viscosity of cow milk was significantly lower than that of the buffalo milk. The surface tension range in goat milk was between 48.50 and 54.28 dynes/cm and the mean value was 51.39 dynes/cm. In cow milk, surface tension range was between 49.72 and 52.32 dyne/cm and the mean value was 51.02 dynes/cm. On the other hand, buffalo milk had surface tension range between 46.41 and 53.27 dynes/cm and the mean value of 49.84 dynes/cm. The goat milk had the highest surface tension, followed by those found in cow and buffalo milk. However, the differences in surface tension of three types of milk studied, *viz*., goat, cow and buffalo milk were found statistically non-significant. The refractive index range in goat milk was between 1.3388 and 1.3460 and the mean value was 1.3424. In cow milk, the range of refractive index was between 1.3390 and 1.3462 and the mean value was 1.3426. On the other hand, in buffalo milk refractive index ranged from 1.3373 to 1.3467 with a mean value of 1.3420. The cow milk had the highest refractive index which was followed by goat milk and the lowest refractive index was found in buffalo milk. However, the differences in refractive index of three types of milk studied, *viz*., goat, cow and buffalo milk were found statistically non-significant. The range of freezing point determined in five replications was −0.550 to −0.468°C and mean value was −0.509°C for goat milk. In cow milk, range of freezing point was −0.564 to −0.516°C with a mean value of −0.540°C. On the other hand, in buffalo milk freezing point ranged between −0.584 and −0.532°C and the mean value was −0.558°C. The goat milk had the lowest freezing point, followed by those found in cow milk and highest freezing point was observed in buffalo milk. The freezing point of goat milk was significantly lower than that of buffalo milk. However, difference in freezing point of goat milk and that of the cow milk was statistically non-significant. Similarly, difference in freezing point of buffalo milk and that of the cow milk was statistically non-significant. The electrical conductivity of goat milk was in the range of 4.70-5.98 mmho with a mean value of 5.34 mmho. In cow milk, range of electrical conductivity was between 4.52 and 4.84 mmho and the mean value was 4.68 mmho. In buffalo milk, electrical conductivity ranged between 4.25 and 5.67 mmho with a mean value of 4.96 mmho. The goat milk had the highest electrical conductivity, which was followed by buffalo milk and lowest electrical conductivity was found in cow milk. However, the differences in electrical conductivity of three type’s milk studied, *viz*., goat, cow and buffalo milk were found statistically non-significant.

**Table-1:** Physicochemical characteristics of goat, cow and buffalo milk.

Types of milk	Parameters						

	Acidity (%LA)	Specific gravity	Viscosity (cp)	Surface tension (dynes/cm)	Refractive index	Freezing point (°C)	Electrical conductivity (mmho)
Goat	0.164±0.032	1.030±0.002	1.53±0.09	51.39±2.89	1.3424±0.0036	−0.509±0.041	5.34±0.64
Cow	0.137±0.022	1.031±0.001	1.59±0.06	51.02±1.3	1.3426±0.0036	−0.540±0.024	4.68±0.16
Buffalo	0.130±0.027	1.032±0.002	1.79±0.11	49.84±3.43	1.3420±0.0047	−0.558±0.026	4.96±0.71
SEM	0.0109	0.00069	0.0296	0.999	0.0015	0.0123	0.20815
CD (0.05)	NS	NS	0.091	3.078	0.0046	0.038	0.64
CV%	17.07	0.15	4.04	4.40	0.25	5.13	9.32

SEM=Standard error of mean, CV=Coefficient of variation, CD=Critical difference, LA=Lactic acid

The collected goat, cow and buffalo milk sample showed negative stability at 68% (v/v) alcohol concentration. Goat milk showed positive alcohol test at 75% (v/v) alcohol concentration. Buffalo and cow milk showed negative alcohol test at 75% (V/V) alcohol concentration. The range of HCT of goat milk samples analyzed was 10.47-11.53 min with a mean value of 11 min at 140°C (Table-2). Similarly, in cow milk samples, HCT ranged between 48.14 and 57.86 min and the mean value was 53. The HCT range in buffalo milk was between 37.49 and 44.51 min and the mean value was 41 min. Thus, cow milk had the highest heat coagulation time (HCT), which was followed by buffalo milk and lowest HCT observed in goat milk. However, the differences in HCT of three types of milk studied, *viz*., goat, cow and buffalo milk were found statistically non-significant. The range of RCT of goat milk samples analyzed was 16.91-32.91 s and with a mean value was 24.91 s at 40°C. Similarly, in cow milk, samples RCT ranged from 21.64 to 33.64 s with a mean value of 27.64 and in buffalo milk it was 28.70-43.70 s with a mean value of 36.20 s. Thus, RCT of goat milk is significantly lower than buffalo milk. Similarly, RCT of buffalo milk is significantly higher than that of cow and goat milk. However, difference in RCT of goat milk and that of the cow milk was statistically non-significant. The rate of acid production by starter culture is mentioned in Table-3. At 0 h, the average % LA of goat milk observed was 0.181. The observations were taken after every hour. After 2, 4, 6, 8, 10, 12 and 14 h, average values of % LA observed were 0.329, 0.394, 0.441, 0.500, 0.581, 0.678, and 0.797. At 0 h, the average % LA of cow milk observed was 0.174. The observations were taken after every hour. After 2, 4, 6, 8, 10, 12, and 14 h, average acidity observed was 0.306%, 0.410%, 0.518%, 0.644%, 0.813%, 0.887%, and 0.968% LA. At 0 h, the average % LA of buffalo milk observed was 0.187. The observations were taken after every hour. After 2, 4, 6, 8, 10, 12 and 14 h, average acidity observed was 0.331%, 0.442%, 0.585%, 0.696%, 0.840%, 0.909%, and 1.002% LA.

**Table-2:** Selected processing-related parameters (HCT and RCT) of goat, cow and buffalo milk.

Types of milk	Parameters	

	HCT (min)	RCT (min)
Goat	11±0.53	24.91±8
Cow	53±4.86	27.64±6
Buffalo	41±3.51	36.20±7.5
SEM	1.315	2.561
CD (0.05)	4.051	7.89
CV%	8.49	19.36

SEM=Standard error of mean, CV=Coefficient of variation, CD: Critical difference, HCT: Heat coagulation time, RCT: Rennet coagulation time

**Table-3:** Rate of acid production by starter culture of goat, cow and buffalo milk.

Period (h)	Acidity (%LA)		

	Goat milk	Cow milk	Buffalo milk
0	0.181	0.174	0.187
2	0.329	0.306	0.331
4	0.394	0.410	0.442
6	0.441	0.518	0.585
8	0.500	0.644	0.696
10	0.581	0.813	0.840
12	0.678	0.887	0.909
14	0.797	0.968	1.002

LA=Lactic acid

The activity of selected enzymes of the samples is mentioned in Table-4. The range of alkaline phosphatase activity determined in five replications was 12.12-19.46 µg p-nitrophenol/ml with a mean value of 15.79 µg p-nitrophenol/ml in goat milk. Similarly, alkaline phosphatase activity range in cow milk was between 29.76 and 31.42 µg p-nitrophenol/ml and the mean value was 30.59 µg p-nitrophenol/ml. On the other hand, alkaline phosphatase activity range in buffalo milk was between 10.29 and 18.63 µg p-nitrophenol/ml and the mean value was 14.46 µg ­p-nitrophenol/ml. Samples of cow milk had the highest alkaline phosphatase activity, which was followed by goat milk and lowest alkaline phosphatase activity was found in buffalo milk. The alkaline phosphatase activity of goat milk was significantly lower than that of the cow milk. However, the difference in alkaline phosphatase activity of goat milk and that of the buffalo milk was statistically non-significant. Alkaline phosphatase activity of buffalo milk was significantly lower than that of the cow milk. The range of catalase activity determined in five replications was 0.58-1.48 units and mean value was 1.03 units in goat milk. Similarly, in cow milk, range of catalase activity was 0.56-1.80 units and mean value was 1.18 units. On the other hand, catalase activity range in buffalo milk was between 0.51 and 1.19 units and the mean value was 0.85 units. Cow milk had the highest catalase activity, which was followed by goat milk and lowest catalase activity was found in buffalo milk. Difference in catalase activity of goat milk and that of the buffalo milk, as well as cow milk, was statistically non-significant. However, difference in catalase activity of cow milk and that of the buffalo milk was statistically non-significant. The range of lipase activity determined in five replications was 0.8-1.6 units and mean value was 1.2 units in goat milk. Similarly, in cow milk, range of lipase activity was 0.8-1.8 units and mean value was 1.3 units. On the other hand, lipase activity range in buffalo milk was between 1.0 and 3.4 units and the mean value was 2.2 units. Goat milk had the lowest lipase activity, which was followed by cow milk and highest lipase activity was found in buffalo milk. However, the differences in lipase activity of three type’s milk studied, *viz*., goat, cow and buffalo milk were found statistically non-significant. The range of proteolytic activity determined in five replications was 386-618 µg tyrosine/ml with a mean value was 502 µg tyrosine/ml in goat milk. Similarly, in cow milk, range of proteolytic activity was 584-728 µg tyrosine/ml with a mean value of 656 µg tyrosine/ml. On the other hand, in buffalo milk, proteolytic activity ranged between 491 and 799 µg/ml with a mean value of 645 µg/ml in samples. Cow milk had the highest proteolytic activity, followed by those found in buffalo and goat milk. The proteolytic activity of goat milk was significantly lower than that of the cow milk. However, difference in proteolytic activity of goat milk and that of the buffalo milk was statistically non-significant. Similarly, difference in proteolytic activity of cow milk and that of the buffalo milk was statistically non-significant.

**Table-4:** Activity of selected enzymes in goat, cow and buffalo milk.

Types of milk	Parameters			

	Alkaline phosphatase (μg p-nitrophenol/ml)	Catalase activity (catalase units)	Lipase activity (units)	Proteolytic activity (µg tyrosine/ml)
Goat	15.79±3.67	1.03±0.45	1.2±0.4	502±116
Cow	30.59±0.83	1.18±0.62	1.3±0.5	656±72
Buffalo	14.46±4.17	0.85±0.34	2.2±1.2	645±154
SEM	1.291	0.186	0.304	46.989
CD (0.05)	3.98	0.573	0.93	145
CV%	14.22	40.86	43.32	17.49

SEM=Standard error of mean, CV=Coefficient of variation, CD=Critical difference

## Discussion

Mahmood and Usman [26] reported the acidity in goat milk as 0.160% LA. Aneja *et al*. [27] found acidity in cow and buffalo milk was 0.15% and 0.16% LA at 20°C, respectively. The data obtained in this study for average acidity of goat milk was very well in agreement with those reported in the literature for milk obtained from goat in India. The data are also in general agreement with those reported for goat milk obtained from exotic goat. Similarly, the data obtained for average acidity of cow milk were also in general agreement with those reported in the literature for cow milk. However, in this study, buffalo milk was found to have lower acidity compared to goat milk as well as cow milk. Mahmood and Usman [26] observed that the specific gravity of Indian goat milk was 1.030. Hanl *et al*. [28] reported the specific gravity range in buffalo milk was between 1.0317 and 1.0380. Park *et al*. [29] reported the specific gravity range in cow milk was between 1.0231 and 1.0398. Imran *et al*. [30] reported viscosity in goat milk was 1.44±0.53 cp. Viscosity of cow and buffalo milk were 1.86 and 2.04 cp, respectively [31]. Gamble *et al*. [24] found that the milk of Toggenburg and Saanen goats had surface tension of 52 dynes/cm. Sindhu [32] reported that buffalo milk had higher surface tension (45.50 dynes/cm at 20°C) compared to cow milk (42.50 dynes/cm at 20°C). The data obtained in this study for average specific gravity, viscosity, surface tension of goat milk was in general agreement with those reported in the literature for milk obtained from goat in India. The data are also in general agreement with those reported for milk obtained from exotic goat. Similarly, the data obtained for average specific gravity, viscosity, surface tension of cow, and buffalo milk were also in general agreement with those reported in the literature for cow and buffalo milk, respectively. Muhammad *et al*. [33] studied refractive index of goat milk collected from different sites of Faisalabad and Pakistan. The mean values reported were 1.3504 and 1.3474. Laxminarayana and Dastur [31] reported that the refractive index in cow and buffalo milk was 1.3338 and 1.3448, respectively. Drackova *et al*. [34] reported freezing point in goat milk was −0.554°C. Laxminarayana and Dastur [31] reported freezing point depression in cow and buffalo milk were −0.540 and −0.560°C, respectively. The data obtained for average refractive index, freezing point of goat milk was in general agreement with those reported in the literature for goat milk. The data obtained for average refractive index, freezing point of cow and buffalo milk were also in general agreement with those reported in the literature for cow and buffalo milk, respectively. Kumar *et al*. [23] reported electrical conductivity in goat milk was between 0.0101 and 0.0188 ohm^−1^ cm^−1^. Aneja *et al*. [27] observed that electrical conductivity in cow and buffalo milk were 4.00-5.50 m mho/cm and 3.22-6.67 m mho/cm, respectively. The data obtained for average electrical conductivity of goat milk was in general agreement with those reported in the literature for goat milk. The data obtained for average electrical conductivity of cow and buffalo milk were also in general agreement with those reported in the literature for cow and buffalo milk, respectively.

Kentaro [35] determined the alcohol stability of fresh milk from cows of various breeds, namely Holstein, Ayrshire, and Guernsey by mixing neutral alcohol of various percentages into the same volume of milk samples. Result showed at 66% alcohol concentration +, ++ test 70% alcohol concentration +, ++ test, 78% alcohol concentration + test, similarly, at 82%, 86%, 90% and 86% alcohol concentration + test. Kakar *et al*. [36] analyzed alcohol tests of buffalo milk samples were positive for control and 10 ppm curd formation of buffalo milk at 6 h and for 20 ppm curd formation of goat milk at 7 h, respectively. Since alcohol stability data of goat milk are similar to reported data in the literature. The results obtained for alcohol stability (ethanol stability) of bovine milk were in accordance with those reported in the literature for cow and buffalo milk, respectively. Palanidorai *et al*. [37] conducted study for goat milk dahi preparation. Fresh goat milk was procured from the farm and was boiled and cooled to room temperature. Mixed lactic cultures were then added at 2% level as the culture were non-specific, and was then incubated at 37±1°C until the acidity reached 0.75-0.80% and were then stored in refrigeration temperature until further use. No reported data are reported in terms of specific value for a rate of acid production by starter culture. According to Muir and Sweetsur [18], goat milk stability at 140°C was 1 min at natural milk pH 6.6 and it showed its maximum stability of 6.8 min at pH 7.2. Gallager and Mulvihill [19] analyzed heat stability of cow milk at 140°C. Results showed maximum heat stability time of cow milk was 30 min at pH 7.1-7.2. Ahmad *et al*. [20] reported HCT of buffalo milk at 140°C was 8 min 48 s. Heat stability of goat milk investigated in this study was found to be lower as compared to reported data. That may be because of compositional difference of the milk specifically salt balance, casein micelle makeup and whey protein content of the milk.

The alkaline phosphatase activity range in goat and cow milks was between 115-1300 and 1800-4750 µg phenol/ml, respectively [38,39]. Sahai [40] analyzed alkaline phosphatase activity in buffalo milk. Alkaline phosphatase activity was 0.1-0.2 Units.mL^−1^. The data obtained in this study for average alkaline phosphatase activity of goat milk were very well in agreement with those reported in the literature for goat milk. However, data obtained in this study for average alkaline phosphatase activity of cow and buffalo milk were also lower than those reported in the literature for cow and buffalo milk, respectively. The overall trend for alkaline phosphatase activity of goat, cow and buffalo milk is also in agreement with that reported in the literature. Catalase activity in goat milk was analyzed by Karmakar *et al*. [22]. It was 30.26 μ mole H_2_O_2_ hydrolyzed min^−1^ ml^−1^ of milk. Catalase activity of mastitic goat milk was 52.85 μ mole H_2_O_2_ hydrolyzed min^−1^ ml^−1^ of milk. Sharma [21] gave the values of catalase activity in cow and buffalo milk in terms of catalase units. The mean value of catalase activity was 0.56 units and 1.08 units for buffalo and cow milk, respectively. Limited data are reported in the ­literature for catalase activity of goat milk. The results obtained in this study for the catalase activity of goat milk are within the range reported in the literature. The results obtained for average catalase activity of cow and buffalo milk were within the range reported in the literature for cow and buffalo milk, respectively. Jandal [41] analyzed lipase activity in goat milk in Iraq and found fresh raw goat milk had 2.76 µEq/ml and raw cow milk had 3.78 µEq/ml activity at 37°C. Sahai [40] analyzed lipase activity in buffalo milk. Lipase activity (Units.mL^−1^) was found to be 0.2-1.1. The results obtained for average lipase activity of goat milk were lower than those reported in the literature for goat milk. The results obtained for average lipase activity of cow milk were lower than those reported in the literature for cow milk. The results obtained for average lipase activity of buffalo milk was higher than those reported in the literature for buffalo milk. Chandan *et al*. [42] detected total protease activity in goat milk. Mean value of 24 milk samples of Camosciata Primiparous goats was 6.17 mU ml^−1^. Sahai [40] analyzed protease activity in buffalo milk. A concentration of protease was 0.8 Units.mL^−1^. Since not much data are reported in the literature for proteolytic activity of goat milk. Less data are reported in the literature for protease activity of buffalo milk. No data are reported in the literature for protease activity of cow milk. The results obtained for average proteolytic activity of goat milk were higher than those reported in the literature for goat milk. The results obtained for average proteolytic activity of buffalo milk was higher than those reported in the literature for buffalo milk.

## Conclusion

It can be concluded from the study that the goat milk has highest acidity, viscosity, electrical conductivity, and surface tension. The goat milk has lowest specific gravity, proteolytic activity freezing point, lipase activity, RCT and HCT compared to cow and buffalo milk. Goat milk had highest refractive index compared to buffalo milk, whereas lowest refractive index compared to cow milk. Goat milk showed positive alcohol test at 75% by volume alcohol concentration. After 14 h when goat milk was observed, it was found that milk became thicker but the curd formed was having very weak consistency. The goat milk has higher alkaline phosphatase activity, catalase activity compared to buffalo milk while it has lower alkaline phosphatase activity, catalase activity compared to cow milk.

## Authors’ Contributions

KDA supervised the experiment. DBP conducted the experiment and the laboratory analysis of the samples. DBK also helped in laboratory analysis of samples and in conduction of experiment. DBP along with AKJ analyzed the data. DBP, DBK, and BMM prepared the manuscript. VBD had helped in statistical analysis of the data. DBP, DBK, BMM, AKJ, and KDA reviewed the manuscript. All authors read and approved the final manuscript.
